# Pattern of Violence Among Healthcare Workers in a Tertiary Care Government Hospital and a Multi-Specialty Private Hospital in Sagar, India: A Cross-Sectional Study

**DOI:** 10.7759/cureus.48231

**Published:** 2023-11-03

**Authors:** Simran Khiyani, Shraddha Mishra, Rupesh Sahu, Abhijit Das, Anuja Pathak

**Affiliations:** 1 Community Medicine, Government Bundelkhand Medical College, Sagar, IND; 2 Community Medicine, Government Chhindwara Institute of Medical Sciences, Chhindwara, IND; 3 Community Medicine, Tripura Medical College and Dr. B.R. Ambedkar Memorial (BRAM) Teaching Hospital, Agartala, IND; 4 Pediatrics, Mahatma Gandhi Memorial Medical College, Indore, IND

**Keywords:** violence against doctors, verbal violence, multi-specialty hospital, tertiary care hospital, medical workplace violence, healthcare worker (hcw)

## Abstract

Background

Workplace violence in hospitals is an occupational hazard that affects healthcare workers (HCWs) negatively in many aspects and causes deterioration of the doctor-patient relationship, resulting in providence of substandard healthcare. This study was conducted to compare the pattern of violence in a tertiary care government teaching hospital and a multi-specialty private trust hospital in Sagar district, Madhya Pradesh, India.

Methodology

After ethical clearance of this cross-sectional, observational study, participants (frontline healthcare workers, including doctors and nurses) were asked about the type, frequency, department, and place of violence, etc., along with its perceived causes, solutions, and arrangements made by hospitals for dealing with it using a pretested, semi-structured questionnaire. Data analysis was performed using IBM SPSS Version 26.0 (IBM Corp., Armonk, NY). Categorical variables were described using frequency and percentages, and inferential analysis was conducted using the chi-square/Fisher's exact test. A P-value of <0.05 was considered statistically significant.

Results

Among the 113 participants, 67 (59.3%) were female, 53 (46.9%) were doctors, and 60 (53.1%) were nurses. The mean age of participants was 30.9±7.3 years. Predominantly verbal, emotional, and physical violence were present in 96.5%, 43.4%, and 6.2% of participants, respectively. Violent incidents against healthcare workers were more frequent in government hospitals as compared to private hospitals. Most healthcare workers (87.6%) tried to resolve violent incidents peacefully, and 1.8% tried to fight back. The most perceived cause of violence in both setups was a lack of morality and literacy among patients and their relatives (i.e., 83.2%), followed by a lack of proper facilities and a lack of trust in healthcare workers.

Conclusion

Both setups faced a substantial amount of violence. The loopholes in both setups, considering resources, security, and other facilities, are clearly visible, and specific steps must be adopted to protect both systems from violence.

## Introduction

Healthcare workers (HCWs) in India include people who help aid the sick directly (doctors and nurses) or indirectly (lab technicians, medical waste handlers, and ward boys) [1]. A tertiary care hospital is a hospital that provides services for the management of complex medical and surgical procedures like treating burns, performing neurosurgery, managing oncological cases, etc. [2]. The multi-specialty hospital includes a hospital that has the capacity to provide treatment that requires personnel from multiple medical specialties.

Workplace violence in the medical field is defined as any action involving abuse (physical, verbal, or emotional), threats, or assaults during the course of duty, posing a significant risk to the safety of healthcare workers [3,4]. Medical workplace violence is a major occupational hazard faced by medical professionals worldwide, and such incidents, which often involve patients, their relatives or attendants, visitors, or mobs, negatively impact the well-being of healthcare staff, patient-physician trust, and patient outcomes [5]. Verbal violence mainly consists of shouting, passing disrespectful remarks, and using offensive language. Emotional violence consists of activities that often have a mental and psychological impact on healthcare workers. Physical violence consists of assault and beating with or without weapons [4,6]. Violence can be caused mainly by patients, patients’ relatives/attendants/visitors, or mobs.

Medical workplace violence is prevalent worldwide. In 2020, healthcare and social assistance workers overall had an incidence rate of 10.3 (out of 10,000 full-time workers) for injuries resulting from assaults and violent acts by other persons; the rate for nursing and personal care facility workers was 21.8 [7]. Violence against doctors and other health workers is common, and its frequency in India appears to be increasing. According to a study by the Indian Medical Association (IMA), 75% of doctors in India have experienced violence at some point in their lives, mostly verbal abuse [8].

The absence of available data regarding the occurrence of violence at the district level or specifically in our chosen study areas hinders efforts to address this issue effectively. Additionally, no prior research has been conducted on the topic in this region. We tried to fill this knowledge gap by shedding light on the patterns of violence in two major hospitals in Sagar. These hospitals serve a large population and hold significant importance within the region, making them suitable for investigation. The research aimed to identify and compare the patterns, perceived causes, and variations of violence among healthcare workers at a tertiary care government hospital and a multi-specialty private trust hospital in Sagar district, Madhya Pradesh, India.

## Materials and methods

Study design, settings, and duration

This was an observational cross-sectional hospital-based study done between June and August 2021. The study was conducted in Sagar, Madhya Pradesh, India. For study purposes, the Government Bundelkhand Medical College (BMC), the only tertiary care government hospital in Sagar, and the Bhagyoday Tirth Trust Hospital (BGTH), the multispecialty private hospital, were chosen. These two hospitals cater to the majority of the population in and around Sagar.

Study population, sample size, and sampling

The study population consisted of healthcare workers who had faced violence in various departments of the two selected hospitals. The sample size was calculated using the formula n=Z2pq/d2, where p=47.02%, which was taken as the prevalence of violence against healthcare workers from a previous study [9]. With a 95% confidence interval, 10% precision, and 10% non-response, the minimum required sample size was 106. Finally, 113 participants were interviewed for the study. Non-probability sampling was done until the required sample size was achieved.

Inclusion and exclusion criteria

Participants were included based on the following criteria: (i) frontline healthcare workers who are likely to come first in contact with the patient; (ii) participants who have faced violence; and (iii) participants who have continuously been a part of the hospital for at least one year.

Participants were excluded based on the following criteria: (i) hospital aides, helpers, lab technicians, and waste handlers; (ii) all those who were not willing to participate in the study and did not provide written consent for the study.

Selection procedure

**Figure 1:**
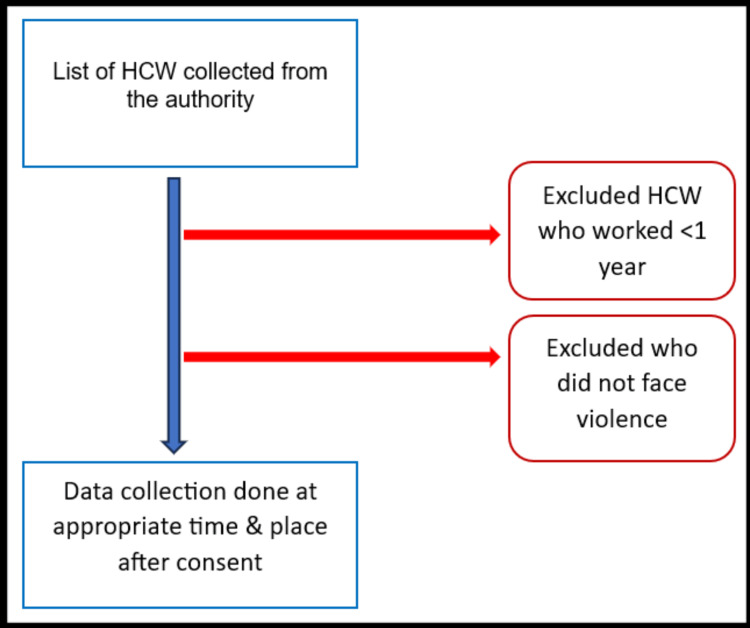
Flowchart showing the selection of participants

The selection process for study participants is shown in Figure 1. Eligible participants were contacted using WhatsApp or a phone call for their appropriate time and place of meeting and were interviewed when they consented.

Data collection tool and study variables

A pre-designed, pre-tested, and semi-structured questionnaire was used for data collection, consisting of closed and open-ended questions. Data were collected on participants' designation, age, gender, duration of working in their respective hospitals, type of violence faced/experienced, frequency of violence, the person causing violence, violence place, damage done to hospital property, shifts in which maximum violence occurs, whether they carry protective equipment for their own safety, perceived causes of violence, and perceived solutions to prevent violence.

Data analysis

Data analysis was performed using IBM Statistical Package for Social Sciences (SPSS) software (IBM Corp., version 26.0, released in 2019; Armonk, NY: IBM Corp.). Categorical variables were described using frequency and percentages. To determine differences between various parameters across different groups, inferential analysis was conducted using the chi-square or Fisher's exact test. A significance level of p<0.05 was used to indicate statistical significance.

Ethical consideration

The Institutional Ethics Committee, Government Bundelkhand Medical College, Sagar, approved this study (IECBMC/2020/08). Permission to conduct the study was obtained from the authorities of the participating hospitals. Participants were explained about the study purpose both in English and Hindi, and written consent was obtained from the participants.

## Results

A total of 113 healthcare workers were selected; among them, the majority (53.9%, 61/113) were from the Bhagyoday Tirth Trust Hospital (BGTH), and the rest (46.1%, 52/113) were from the Government BMC. Among the participants in BGTH, 29 were doctors and 32 were nurses. In contrast, the participants in BMC consisted of 24 doctors and 28 nurses. The mean age of all participants was 30.9±7.3 years. Specifically, the mean age for BGTH participants was 31.2±6.3 years, while BMC participants had a mean age of 30.5±8.4 years. The median (IQR) duration of the working year for the participants of BGTH was 3.0 (2.0-5.0) years, and that of BMC was 3.0 (1.5-5.5) years. The rest of the characteristics are described in Table 1.

**Table 1:** Basic characteristics of participants

Variables	Sub-category	Overall (n=113)		BGTH (n=61)		BMC (n=52)	
		Frequency	Percentage	Frequency	Percentage	Frequency	Percentage
Gender	Male	46	40.7	31	50.9	15	28.8
	Female	67	59.3	30	49.1	37	71.2
Age (in years)	20–29	56	49.6	29	47.6	27	51.9
	30–39	42	37.2	25	41.0	17	32.7
	40–49	11	9.7	6	9.8	5	9.6
	50–59	4	3.5	1	1.6	3	5.8
Designation of doctors	Senior doctor	24	21.3	13	21.3	11	21.1
	Senior resident	4	3.5	3	4.9	1	1.9
	Junior resident	25	22.1	13	21.3	12	23.1
Designation of nurses	Nursing sister	5	4.4	4	6.6	1	1.9
	Nursing staff	48	42.5	28	45.9	20	38.5
	Nursing student	7	6.2	0	0	7	13.5

**Figure 2:**
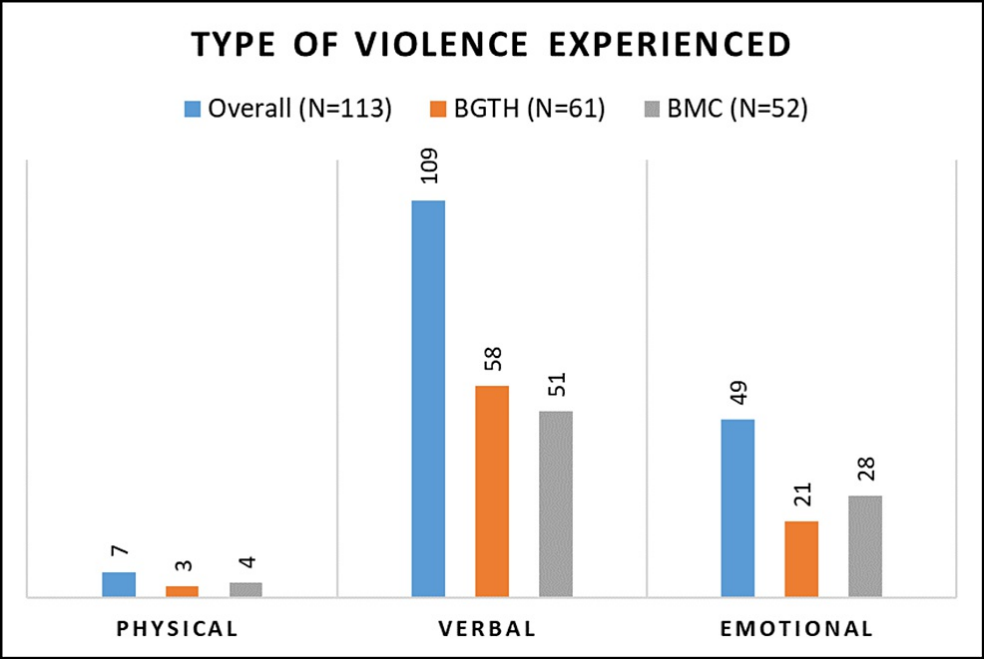
Distribution of participants according to the type of violence experienced

Verbal violence was the most common form of violence reported by the participants from both setups, i.e., 98.1% (51/52) and 95.1% (58/61) in government and private, respectively (Figure 2). When faced with violence, most participants tried to resolve the matter peacefully, i.e., 90.2% and 84.6% in private and government setups, respectively. Taking no action for the incident was more common in private setups (6.5%) (Table 2).

**Table 2:** Distribution according to the option chosen for coping with the violence This table contains multiple responses; percentages are shown based on “n”

Variables	Overall (n=113)		BGTH (n=61)		BMC (n=52)	
	Frequency	Percentage	Frequency	Percentage	Frequency	Percentage
Take no action	6	5.3	4	6.5	2	3.8
Try to resolve peacefully	99	87.6	55	90.2	44	84.6
Try to fight back	2	1.8	0	0	2	3.8
Try to call for help	33	29.2	3	4.9	30	57.7
Report to higher authorities	34	30.1	3	4.9	31	59.6
Report to police	6	5.3	1	1.6	5	9.6

The patient's relatives and mob were mostly responsible for causing maximum violence in both setups, i.e., 76.5% and 51.7% in the government and private setups, respectively. Meanwhile, patients themselves were more commonly involved in verbal and emotional violence. The violence faced by every participant was inside the hospital; in addition, some participants faced violence outside the hospital (9.6% in BMC and 8.1% in BGTH). Damage done to hospital properties during acts of violence was found to be almost the same for both setups, i.e., 25% for the government setup and 24.6% for the private setup. Most violent incidents occurred during night shifts, i.e., 36.5% and 34.4% for government and private setups, respectively. The majority of participants did not carry any protective equipment with them, but carrying a knife, pepper spray, and scissors was reported by 7.69% of government setup participants. Most participants from both setups, i.e., 49.2% (30/61) from BGTH and 59.6% (31/52) from BMC, identified the emergency department as the primary location where most incidents of violence took place. Table 3 displays the satisfaction level of participants from both hospitals regarding their arrangements to tackle violence, and it was found to be statistically significant (p<0.05).

**Table 3:** Distribution of participants according to satisfaction with the hospital’s arrangement for tackling violence *Statistically significant

Variables	Sub-category	Overall (n=113)		BGTH (n=61)		BMC (n=52)		p-value
		Frequency	Percentage	Frequency	Percentage	Frequency	Percentage	
Security check for people entering the hospital	Satisfied	31	27.4	24	39.3	7	13.4	0.002*
	Moderately satisfied	20	17.7	13	21.4	7	13.4	
	Not satisfied	62	54.9	24	39.3	38	73.2	
CCTV installation in the hospital	Satisfied	43	38.0	37	60.7	6	11.5	<0.001*
	Moderately satisfied	22	19.5	5	8.2	17	32.7	
	Not satisfied	48	42.5	19	31.1	29	55.8	
Adequacy of security workforce recruitment	Satisfied	33	29.2	27	44.3	6	11.5	<0.001*
	Moderately satisfied	22	19.5	8	13.1	14	26.9	
	Not satisfied	58	51.3	26	42.6	32	61.6	
Amount and type of weaponry available for security	Satisfied	23	20.4	20	32.8	3	5.8	0.002*
	Moderately satisfied	13	11.5	6	9.8	7	13.5	
	Not satisfied	77	68.1	35	57.4	42	80.7	
Actions taken against the violent incident	Satisfied	54	47.8	37	60.7	17	32.7	0.005*
	Moderately satisfied	22	19.5	6	9.8	16	30.8	
	Not satisfied	37	32.7	18	29.5	19	36.5	

**Table 4:** Distribution according to the perceived cause of violence This table contains multiple responses; percentages are shown based on “n”; *statistically significant

Perceived causes	Overall (n=113)		BGTH (n=61)		BMC (n=52)		p-value
	Frequency	Percentage	Frequency	Percentage	Frequency	Percentage	
Lack of trust in healthcare workers	41	36.3	15	24.6	26	50.0	0.005*
Delay in providing treatment	25	22.1	18	29.5	7	13.4	0.043*
Lack of proper facilities	52	46.0	20	32.8	32	61.5	0.003*
Lack of literacy and mortality among patients and their relatives	94	83.2	46	75.4	48	92.3	0.018*
Lack of coordination between patient and healthcare workers	31	27.4	11	18.0	20	38.4	0.017*
Unavailability of healthcare workers	25	22.1	18	29.5	7	13.4	0.043*
Misbehavior/improper response by healthcare workers	10	8.8	6	9.8	4	7.6	0.952

Lack of literacy and mortality among patients and their relatives was the commonest perceived cause for violence in private (BGTH) and government (BMC) setups, i.e., 75.40% and 92.3%, respectively (Table 4). Participants also cited that lack of trust and delayed treatment are associated with violence in government hospitals, and overpriced facilities are responsible for violence in private hospitals. Table 5 shows the distribution of solutions for preventing violence as perceived by the HCWs, which shows that limiting the number of people accompanying the patient was the commonest perceived solution for the prevention of violence by participants of both private (BGTH) and government (BMC) setups, i.e., 78.68% and 82.69%, respectively.

**Table 5:** Distribution according to the perceived solutions chosen to prevent violence This table contains multiple responses; percentages are shown based on “n”; * statistically significant

Perceived solutions	Overall (n=113)		BGTH (n=61)		BMC (n=52)		p-value
	Frequency	Percentage	Frequency	Percentage	Frequency	Percentage	
Increasing the number of security personnel	69	61.1	33	54.1	36	69.2	0.105
Installation of panic buttons	59	52.2	32	52.5	27	51.9	0.955
Improving communication between doctors and patients	31	27.4	17	27.9	14	26.9	0.006*
Stricter laws and actions must be taken by the administration against acts of violence/abuse	63	55.8	27	44.3	36	69.2	0.008*
Conducting mock drills of violent episodes as practice to handle violence	21	18.6	13	21.3	8	15.4	0.434
Healthcare workers should behave politely (acquire soft skills)	31	27.4	20	32.8	11	21.1	0.176
Limiting the number of people accompanying the patient	91	80.5	48	78.7	43	82.7	0.605
Improvisation of hospital facilities	10	8.8	7	11.7	3	5.7	0.468

## Discussion

This study aimed to identify and compare the patterns, perceived causes, and variations of violence among healthcare workers of a tertiary care government hospital and a multi-specialty private trust hospital in Sagar district, Madhya Pradesh, India, in a calculated sample of 113. Though very few studies have been conducted on the issue of the comparison of workplace violence in a tertiary care government setup and a multi-specialty private setup, to the best of our knowledge, this is the first study that addressed this issue in the Sagar district of Madhya Pradesh.

Type and pattern of violence

Results of this study showed that verbal violence (96.5%) was the commonest form of violence faced by the participants from both the government (98.1%) and private setups (95.1%), followed by emotional violence and physical violence; these results are more than what is reported in a multicenter study that reports 81.1% of verbal abuse in emergency departments [10]. Another Malaysian cross-sectional study conducted in two departments reported around 70% of verbal abuse among HCWs. The difference present is partly methodological, as these studies included random HCWs, whereas our study included only those who suffered violence [11], but it is evident that verbal violence is the most common. The majority of our study participants, i.e., who faced violence, were in the age group of 20-29 years (49.6%, 56/113), followed by 30-39 years (37.2%, 42/113). These data also indicate that younger healthcare workers were more prone to workplace violence than their older counterparts in both setups, which aligns with Malaysian and other studies that revealed similar patterns [11-13]. However, it contradicts the observations made by Alsaleem et al.; in their study, they reported 3% more violence among older HCWs as compared to younger ones [14]. It was also found that more work experience is linked with a lesser risk of facing violence, similar to a previous study by Lepping et al. [15]. Younger people may be perceived as having less experience, less education, and an inability to deal with violence. It was also observed that older healthcare workers acquire experience in dealing with patients and their relatives and help in avoiding such incidents.

Similar to the results of Pinar et al., our results also showed a higher occurrence of violence in government hospitals than in private ones [16]. This could be related to the fact that government hospitals are overcrowded, understaffed, and underresourced as compared to private hospitals; however, in the present study, participants do not think that being understaffed is a reason for violence [17]. Moreover, government hospitals deal with large populations, especially rural populations, who are often illiterate and have less medical knowledge. Among various departments of hospitals, emergency departments were more vulnerable to violent incidents in both government and private settings, which coincides with other previous studies [11,18,19,20]. These findings may be attributed to the need for spontaneous coordination between multiple medical specialties in emergency departments, leading to longer waiting times and causing stress for patients, relatives, and healthcare workers. Consequently, this can also result in a perception of inadequate treatment by patients' relatives [20].

Most violent incidents occurred during the night shifts, since night shifts are associated with less security, less availability of medical staff, and hence more vulnerability for such incidents, followed by morning and evening shifts in both setups, which is consistent with the findings of a previous study by Davey et al. [10]. However, this contrasts with the study by Li et al., which reported that most violent incidents happen during the day shift [18]. The day's shifts are associated with increased patient load, longer waiting times, and delayed treatment during outpatient clinic hours, contributing to the higher occurrence of violent incidents. Most of the violent incidents took place inside the workplace in both setups. Outside the workplace, violence was generally perpetrated by people who were assumed to withhold power, were strong financially, and often threatened the HCWs, similar to the results found by Jakobsson et al. [21].

Perceived causes

Though lack of morality and literacy among patients and patients’ relatives was the most commonly perceived cause, followed by lack of proper facilities in both setups, the proportion of this opinion was higher in the government setup and was statistically significantly different as well (Table 4). A qualitative study conducted by Davey et al. suggests a long waiting period/crowd as the commonest reason, followed by a lack of morality/literacy [10]. Moreover, lack of trust and delayed treatment are associated with violence in government hospitals, and overpriced facilities are responsible for violence in private hospitals. It is interesting to note that in a study conducted from the patient's relatives’ perspective, misinformation about their patient, unjust treatment, ignorance, and longer waiting times were cited as primary causes of violence [20]. However, HCWs in our study don’t think so. The reasons for violence differ from HCWs' perspective and patients'/relatives' perspective. This may be a limitation in controlling the violence.

Perceived solutions

According to participants from both setups, the perceived solutions to prevent the violence were limiting the number of people accompanying the patient (80.5%), followed by increasing the number of security personnel (61.1%), installing panic buttons (52.2%), improving communication between doctor and patient, and HCWs behaving politely (27.4%). However, more participants from the government setup suggested that stricter laws and actions by the administration and other hospital administration-based interventions are required to prevent violence. Studies by Kumar et al. and Davey et al. suggest similar solutions [9,10]. Surprisingly, HCWs from both setups did not give priority to improving communication between HCWs and patients for avoiding violence, although it was a major reason for violence in the study conducted by Bingöl and İnce on the patients’ relatives perspectives and other studies [19,20].

Satisfaction with hospitals arrangement for tackling violence and coping mechanisms

The HCWs from the government setup seem more dissatisfied with the hospital efforts, such as security checks for the people entering hospital premises, the adequacy of security workforce recruitment and weaponry available for them, or the actions taken against the violent incident, all of which were found to be statistically significant (p<0.05) among HCWs of both setups. As pointed out by this study, among the actions taken to cope with the violent incidents, most healthcare workers tried to resolve the violent situation peacefully in both setups. At the same time, healthcare workers in government setups tried calling for security and reporting the incident to higher authorities more as compared to private setup healthcare workers. Other methods for coping with these violent incidents were changing shifts, departments, units, and duties and carrying any protective equipment with them. Four healthcare workers in the government setup reported carrying a knife, scissors, and pepper spray with them. This points to the negligence of authorities, which renders workers in such a helpless position that they think of resorting to such options for self-defence. Debnath et al. and Anand et al., in their studies, found that very few of the victims of workplace violence chose to report these incidents to their superiors. Those who refrained from reporting often cited reasons such as perceiving it as a futile and time-consuming process, a lack of support from their organization, and the absence of proper channels for reporting such occurrences [6,22]. This highlights the need to encourage reportage of violence among afflicted workers and to develop institutional mechanisms for speedy measures to avoid such events.

Strengths and limitations

This study provides direct, actionable points for solutions to the violence as perceived by the healthcare workers themselves. However, this study has its limitations, such as focusing only on those healthcare workers who have faced violence; hence, it cannot comment on the prevalence of violence. This study asked the participants to recall the incidences of violence, which makes them susceptible to recall bias. Apart from this, ours was a time-bound project; therefore, only one hospital from both sectors could be involved, hence the results do not claim to be representative of government or private hospitals. Similar studies with more hospitals from both sectors may yield better results.

## Conclusions

Healthcare workers face different forms of violence across different levels of the healthcare system. To address this issue, it is crucial to implement necessary interventions, including strict enforcement of existing laws in a fair and appropriate manner. Incorporating mock drills and training on patient interaction and communication into the curriculum of healthcare students is essential. Future research should focus on investigating the underlying causes that drive patients and visitors to resort to violence, as well as examining the effects of such incidents on healthcare workers. Additionally, studies should develop strategies to promote awareness among the general population, aiming to reduce the occurrence of violent acts.
